# Supplementary figures and images for: The Reparative Function of MMP13 in Tertiary Reactionary Dentinogenesis after Tooth Injury
Source: Int J Mol Sci. 2024 Jan 10;25(2):875. doi: 10.3390/ijms25020875 (PMC10815342; doi:10.3390/ijms25020875)

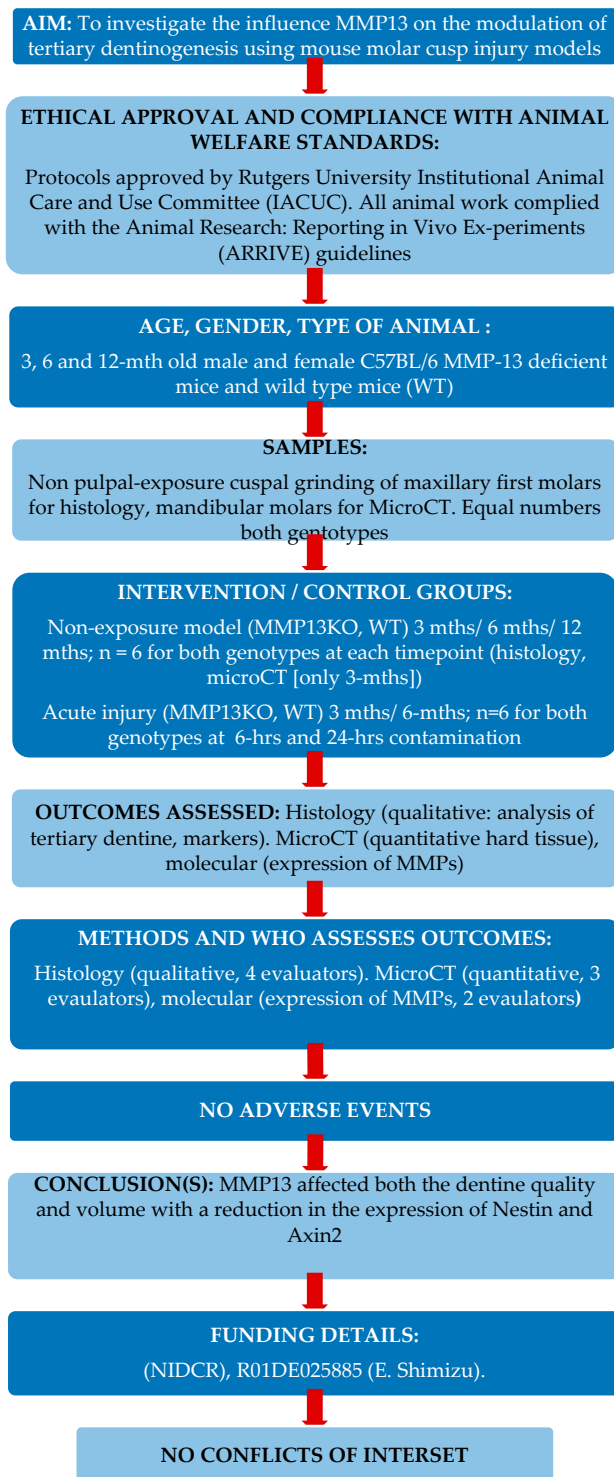

**Supplementary Figure S1:** Experimental flowchart

Supplement: Supplementary file 1 [file ijms-25-00875-s001.zip › ijms-2776233-supplementary.pdf]
